# Prescribing patterns for hypertensive disorders of pregnancy in low- and middle-income countries: a systematic review

**DOI:** 10.1186/s12884-026-08971-z

**Published:** 2026-03-28

**Authors:** Hammad Atif Irshad, Muhammad Ali Akbar Khan, Ayesha Yaseen, Bilal Ahmed Lodhi, Fatima Abdullah, Sarah Mansoor, Zohra S. Lassi

**Affiliations:** 1 Medical College, Aga Khan University, Stadium Road, P.O. Box 3500, Karachi, 74800 Sindh Pakistan; 2 Department of Family Medicine, Aga Khan University Hospital, Karachi, Pakistan; 3 School of Public Health, Faculty of Health and Medical Sciences, The University of Adelaide, Adelaide, Australia; 4 Robinson Research Institute, The University of Adelaide, Adelaide, Australia

**Keywords:** Prescribing patterns, Anti-hypertensives, Low-Middle Income countries (LMICs), Gestational Hypertension, Pre-eclampsia

## Abstract

**Background:**

Hypertensive Disorders of Pregnancy (HDPs), encompassing gestational hypertension, pre-eclampsia, and eclampsia, affect approximately 18 million pregnancies globally. While international guidelines specify management for these disorders, their implementation in low-and middle-income countries (LMICs) is unassessed. Therefore, this systematic review aims to assess the prescribing patterns for HDPs to gauge suitable recommendations for practitioners in LMICs.

**Methods:**

A systematic review was conducted using PubMed, CINAHL, Scopus and Google Scholar using relevant terms for HDPs, prescribing patterns and LMICs. Observational studies published between January 2000 and November 2023 reporting management of HDPs in LMICs were included. The quality of the articles was screened using the National Heart Lung and Blood Institute (NHLBI) quality assessment tool.

**Results:**

A total of 54 studies comprising 14,598 HDP cases from 17 LMICs were included. Of these studies, 18 reported prescriptions for gestational hypertension, in which, calcium channel blockers were reported to be prescribed in all, while the frequency of other prescribed medications included labetalol (89.0%) and methyldopa (66.7%). 41 studies reported medications for pre-eclampsia which included magnesium sulfate—MgSO_4_ (68.3%), nifedipine (61.0%), methyldopa (48.8%) and labetalol (43.9%) prescriptions. A majority of the 28 studies reporting on eclampsia mentioned the use of MgSO_4_, while 33% prescribed diazepam, and phenytoin was prescribed in 15% of the studies.

**Conclusion:**

Majority of LMICs tend to prescribe the established first line medications for HDPs. Slight differences are seen in combinations, likely due to use of different guidelines and medication availability. Standardization of institutional guidelines and strategies to increase adherence to guidelines across LMICs are crucial for improved outcomes and safer prescribing patterns among physicians. Several studies report an absence of institutional guidelines, while practices differ from international guidelines, necessitating the need for standardization of prescribing patterns across resource-limited settings.

## Introduction

According to the World Health Organization (WHO), around 18 million women worldwide are diagnosed with hypertensive disorders of pregnancy (HDPs), attributing 14% of all global maternal deaths. While high-income countries (HICs) have made progress in mitigating the burden of these disorders, women in low- and middle-income countries (LMICs) are still disproportionately affected [1]. Moreover, the years of life lost to HDPs in LMICs are approximately 36 times those in HICs, and a direct correlation has been reported between disease burden and low human development indices [1].

Pharmacologic treatment along with regular blood pressure monitoring and supportive care, are a mainstay for managing HDPs [2]. Given the importance of pharmacologic management, established international guidelines exist to help guide the choice of treatment [3–10]. However, these guidelines have been formed mainly in HICs, and various factors limit their implementation in LMICs. Some international consensus guidelines have been formed but their use is also not applicable in all settings [11]. In circumstances where guidelines have been specified, providers may not be able to prescribe the first-line agent due to its non-availability. Compounding this issue is the unavailability of medications, or one of its formulations in the market; or the presence of a monopoly that renders the medication too expensive [12]. This is exemplified by the unavailability of extended-release formulations of nifedipine in the Indonesian market despite its role in treating gestational hypertension [13]. Similar problems concerning the utilization of MgSO_4_ for the management of severe pre-eclampsia and eclampsia have also been reported, including inadequate inventory management and the absence of sufficient stocks [14]. However, even in situations where medications are available, a lack of adequate training and provider knowledge is a factor that can directly influence the timing and choice of medication prescribed, especially in primary or secondary care settings [15].

Literature further indicates that anti-hypertensives tend to cost higher in LMICs, and the scarcity of essential medications serves as a hindrance to the provision of care [16]. Excessive costs can impact the management of HDP in multiple ways. For many patients, arranging the necessary funds to afford antihypertensive medications may be difficult, with literature citing financial constraints as a major challenge in LMICs such as Ghana [17]. Prescribers may therefore not be able to provide the required management due to factors beyond their control [18].

Therefore, a critical assessment of the current prescribing patterns is essential to determine the necessary revisions so current guidelines are better applicable for practice in LMICs. While prescribing patterns of antihypertensives for hypertension have been systematically explored in LMICs [19], no such study exists for HDPs. Therefore, the objective of our review is to evaluate the prescribing patterns of medications for gestational hypertension, pre-eclampsia, and eclampsia in LMICs.

## Methods

This study is registered in the International Prospective Register of Systematic Reviews (PROSPERO ID: CRD42023493336) and was conducted in compliance with the Preferred Reporting Items for Systematic Reviews and Meta-Analyses (PRISMA) statement [20].

### Search strategy and information sources

Our search approach involved using controlled vocabulary, in the form of MeSH terms, alongside free text. To compile a comprehensive set of keywords, we drew from relevant studies and reviews, which encompassed themes like "Prescribing patterns," "hypertensive disorders of pregnancy" and "developing countries," along with their suitable synonyms. The detailed search strategy can be found in Supplementary Table 1.

To ensure comprehensive coverage, we conducted separate searches across four databases (PubMed, CINAHL, Scopus, and Google Scholar) for LMICs listed by the World Bank [21]. Furthermore, we conducted manual searches by examining bibliographies and cross-references in selected publications, aiming to identify relevant articles.

### Eligibility criteria

#### Inclusion criteria

Observational studies from LMICs, according to the World Bank, published between January 2000 and November 2023 with pregnant women that experienced hypertension manifesting de novo at or after 20 weeks of pregnancy requiring pharmacological intervention were included in the study [21]. This consisted of the following categories: gestational hypertension, pre-eclampsia, and eclampsia [10, 22, 23]. Moreover, any medications used needed to be specified.

#### Exclusion criteria

Studies were excluded if they were conducted in a HIC and did not include pharmacological interventions for HDPs. Studies in non-English languages or falling outside the specified time frame were also excluded.

### Operational definitions

#### Gestational hypertension

Hypertension arising de novo after 20 weeks of gestation with the absence of proteinuria and without any biochemical/hematological abnormalities [10].

#### Pre-eclampsia

De novo hypertension after 20 weeks gestation with either proteinuria and/or evidence of organ dysfunction [10].

#### Eclampsia

Seizure during pregnancy in patients with hypertension not due to any other neurological or metabolic cause [24].

### Study selection

Following the completion of the database search, the identified studies underwent a relevance assessment. Articles focusing on prescription/drug usage patterns underwent primary screening to determine their eligibility for full-text screening. The Rayyan software for systematic reviews facilitated this screening process [25]. The primary screening involved two independent reviewers (MAAK and AY) who assessed the titles and abstracts for relevance. After title and abstract screening, selected papers underwent full-text screening. Two reviewers (BAL and FA) participated in the full-text screening process and any disagreements between the authors were settled by consensus.

### Data extraction

During the independent data collection process, two investigators (BAL and FA) autonomously extracted pertinent information from the studies using a pre-prepared form. To streamline the data collection, Microsoft Excel was employed as a facilitative tool. The extracted variables encompassed the study's title, authors, publication year, study design, sample size, country of origin, type of HDP, and the prescribed medication.

Additionally, secondary variables such as the trimester when the medication was prescribed, demographic factors such as age and socio-economic status (if provided), and the level of care (primary, secondary, or tertiary), were also extracted. Specifically for the treatment of the eclampsia regimens, details such as route of administration, loading dose, maintenance dose, and monitoring parameters of magnesium sulfate were also extracted, if provided. The results from each included study were compiled into an Excel spreadsheet.

### Assessment of risk of bias

To ensure a thorough evaluation of each study's internal validity and risk of bias, two independent reviewers (AY and BAL) employed the National Heart Lung and Blood Institute (NHLBI) quality assessment tool, specifically tailored for observational cohort and cross-sectional studies [26]. The reviewers assessed various components of the scale, assigning responses such as "Yes," "No," "Not Applicable," or "Not recorded" based on their evaluation of each criterion. These responses guided the overall rating of the study's quality which was categorized as good, fair, or poor. In cases of disagreements over the rating, a third reviewer (HAI) was consulted. The individual overall ratings from each reviewer were duly reported.

### Data synthesis

Detailed information on the medication was also extracted, including the class and name of the prescribed medication and the proportion of medication prescribed. The percentage of medications prescribed or used in these studies was tabulated according to the hypertensive disorder. The prescribing patterns were then compared to international guidelines which outlined antihypertensive therapy [27], as seen in Table 1.

**Table 1:** International guidelines used for the management of HDPs

International Guidelines	Country of origin
Royal College of Obstetricians and Gynaecologists (RCOG)	United Kingdom
American Congress of Obstetricians and Gynecologists (ACOG) [28]	United States
Dutch Society of Obstetrics and Gynaecology (NVOG) [8]	Netherlands
National Institute for Health Care Excellence (NICE) guidelines [3]	United Kingdom
Queensland Maternity and Neonatal Clinical Guidelines Program (QLD) [8, 29]	Australia
Society of Obstetricians and Gynaecologists of Canada (SOGC) [7]	Canada
The ISSHP recommendation [5, 6, 10]	International consensus
The Society of Obstetric Medicine of Australia and New Zealand (SOMANZ) guideline [30]	Australia
WHO Recommendations [4, 9]	International consensus

## Results

### Study selection

**Fig. 1:**
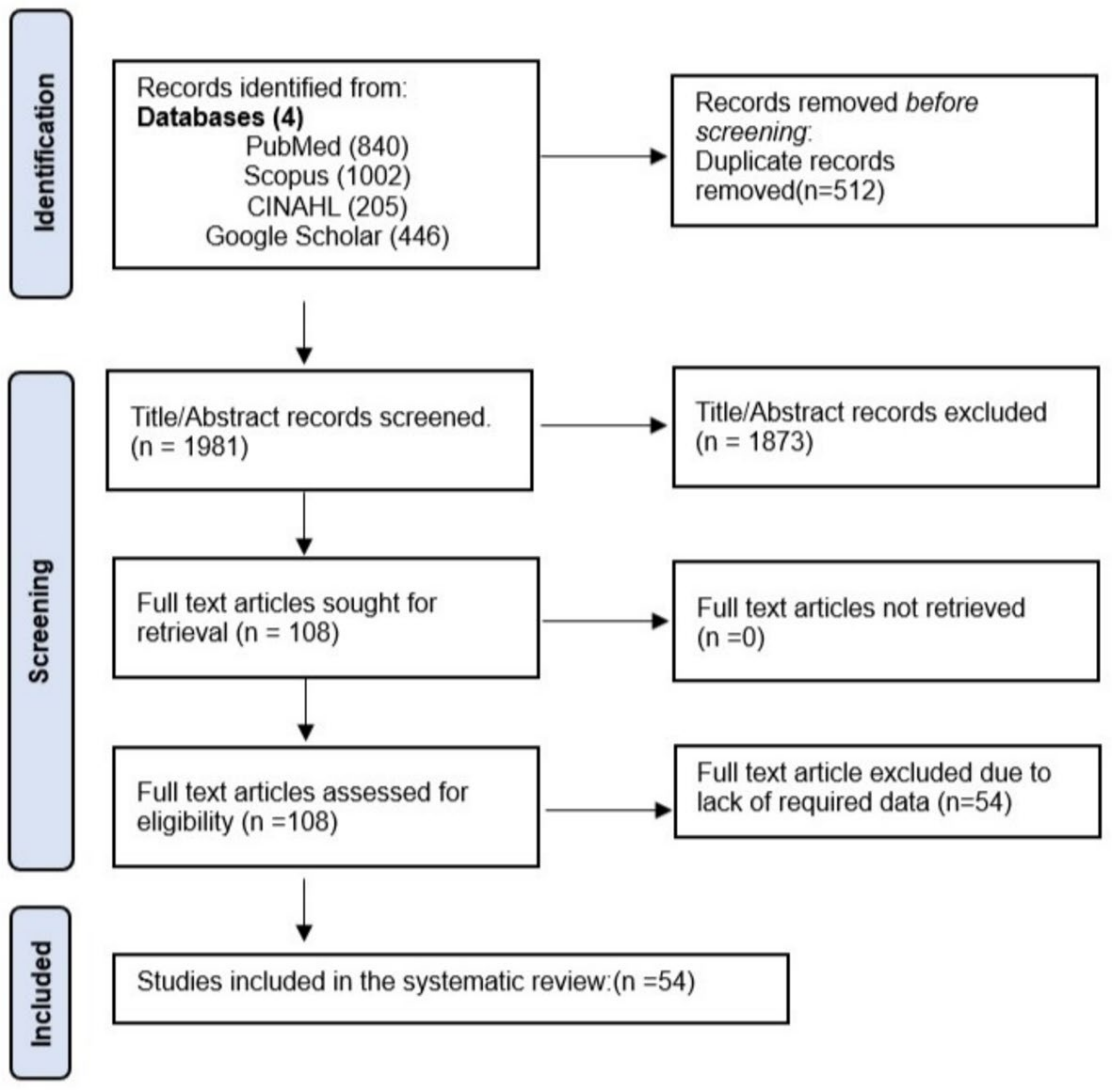
PRISMA flowchart

The research process yielded 2,493 records from various databases. After screening, including the removal of duplicates and assessment of titles and abstracts, a total of 108 full text studies were evaluated for eligibility. Among these, 54 articles were excluded because they did not contain data on medications. The PRISMA flowchart can be seen in Fig. 1. In the end, 54 independent records from 17 different countries met the specified inclusion criteria.

### Study characteristics

**Fig. 2:**
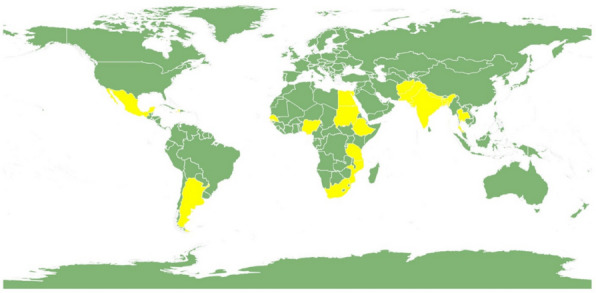
Map with included countries highlighted

Figure 2 displays a map of the included countries. Among included studies, 23 studies were from India [31–53], seven from Nigeria [54–60], five from Ethiopia [61–65], three from Afghanistan [15, 66, 67], two each from South Africa [68, 69], Bangladesh [70, 71], Tanzania [72, 73] and one each from Argentina [74], Egypt [75], Senegal [76], Nepal [77], Sudan [78], Haiti [79], Pakistan [80], and two studies involving multiple countries (Mexico and Thailand in one and India, Pakistan and Mozambique in the other) [81, 82].

Regarding study design, the included studies were categorized as follows: 23 were retrospective [35–38, 42, 43, 45, 50, 51, 53, 54, 58–60, 62, 64, 65, 67, 70, 72, 76, 81, 83], 17 were cross-sectional [15, 33, 44, 48, 49, 52, 56, 57, 61, 66, 68, 71, 73–75, 77, 78, 82], one mixed-methods [57] design; 12 were prospective [31, 32, 34, 39–41, 44, 46, 47, 55, 63, 69, 80] and one study employed both prospective and retrospective elements [79]. The characteristics of the included studies can be seen in Table 2**.**

**Table 2:** Study characteristics

First Author et al	Year of Publication	Country	Study Design	Study Setting
Khedun et al. [68]	2000	South Africa	Cross-Sectional	N/A
Ikechebulu et al. [60]	2002	Nigeria	Retrospective	Tertiary Care
Shaheen et al. [80]	2003	Pakistan	Prospective	Tertiary care hospital
Lumbiganon et al. [81]	2007	Mexico and Thailand	Retrospective	Secondary Care
Uchenna et al. [54]	2007	Nigeria	Retrospective	Secondary and Tertiary Care
Kidanto et al. [73]	2009	Tanzania	Prospective/Audit	Tertiary Care
Tukur et al. [84]	2009	Nigeria	Retrospective	Tertiary Care
Abd El Aal et al. [75]	2012	Egypt	Prospective + Cross-sectional	Tertiary Care
Sariem et al. [59]	2012	Nigeria	Retrospective	Tertiary Care
Chaturvedi et al. [49]	2013	India	Cross-Sectional	Secondary and Tertiary Care
Hooli et al. [50]	2013	India	Retrospective	Tertiary Care
Kim et al. [15]	2013	Afghanistan	Cross-sectional	All levels of Care
Kumar T et al. [36]	2013	India	Retrospective	Tertiary Care
Cormick et al. [74]	2014	Argentina	Cross-sectional	Tertiary Care
Kumar et al. [34]	2014	India	Prospective	Tertiary Care
Sajith et al. [32]	2014	India	Prospective	Tertiary Care
Ahadi et al. [67]	2015	Afghanistan	Retrospective	Tertiary Care
Oguntunde et al. [57]	2015	Nigeria	Mixed-Methods	Primary and Secondary Care
Devkota et al. [77]	2016	Nepal	Cross-sectional	Tertiary Care
Minimal. P.V et al. [85]	2016	India	Prospective	Tertiary Care
Yamakabanardi et al. [35]	2016	India	Retrospective	Tertiary Care
Divyashree et al. [37]	2017	India	Retrospective	Tertiary Care
Patel et al. [44]	2017	India	Cross-Sectional	Tertiary Care
Yousef et al. [78]	2017	Sudan	Cross-Sectional	Tertiary Care
Ali et al. [83]	2018	Pakistan	Retrospective	Tertiary Care
Katageri et al. [52]	2018	India	Cross-Sectional	Primary and Secondary Care
Lahamate et al. [43]	2018	India	Retrospective	Tertiary Care
N Vijayan et al. [51]	2018	India	Retrospective	Tertiary Care
Ansari et al. [66]	2019	Afghanistan	Cross-Sectional	All levels of Care
Maaløe1 et al. [72]	2019	Tanzania	Retrospective	Tertiary Care
Prabahar et al. [42]	2019	India	Retrospective	Tertiary Care
Selvi et al. [47]	2019	India	Prospective	Secondary Care
Shekhar et al. [38]	2019	India	Retrospective	Tertiary Care
Toure et al. [76]	2019	Senegal	Retrospective	Tertiary Care
Williams et al. [70]	2019	Bangladesh	Retrospective	Primary and Secondary Care
Jacob et al. [48]	2020	India	A descriptive and correlational study,	Tertiary Care
Kanafileskookalayeh et al. [40]	2020	India	Prospective	Tertiary Care
Ngene et al. [69]	2020	South Africa	Prospective	Tertiary Care
Obadeji et al. [56]	2020	Nigeria	cross-sectional study	Tertiary Care
Suthar et al. [33]	2020	India	Cross-Sectional	Tertiary Care
Makiabadi et al. [31]	2021	India	Prospective	Tertiary Care
Billah et al. [71]	2021	Bangladesh	Cross-sectional	Primary Care
Bone et al. [82]	2021	India, Pakistan and Mozambique	Cross-Sectional	Primary Care
Getaneh et al. [62]	2021	Ethiopia	Retrospective	Secondary Care
Godana et al. [63]	2021	Ethiopia	Prospective	Tertiary Care
Malhamé et al. [79]	2021	Haiti	Prospective and Retrospective	Tertiary Care
Oyeneyin et al. [55]	2021	Nigeria	Prospective	Tertiary Care
Tlaye et al. [65]	2021	Ethiopia	Retrospective	Tertiary Care
Meazaw et al. [64]	2022	Ethiopia	Retrospective	Secondary and Tertiary Care
Panchal et al. [41]	2022	India	Prospective	Tertiary Care
Pawar et al. [45]	2022	India	Retrospective	Tertiary Care
Seetharamaiah et al. [53]	2022	India	Retrospective	Tertiary Care
Syoum et al. [61]	2022	Ethiopia	Cross-sectional	Secondary Care
Blessy et al. [39]	2022	India	Prospective	Tertiary Care
N/A: Not Applicable				

Among included studies, 18 studies reported on medications for GH, 41 on PE, and 31 for eclampsia. Patient characteristics are described in Supplementary Table 2. The patients’ ages ranged between 14 and 45 years [75, 76], with the majority falling within the 20 to 35 age range across 10 studies [36, 41, 43, 46, 53, 63, 67, 69, 77, 78]. Information regarding obstetric was found in 34 studies. Of these articles, 12 mentioned parities [34, 44, 51, 55, 61, 63, 64, 67, 72–74, 76], with 8 of them reporting that the majority of their patients fell under the category of NP (nulliparous) [44, 51, 55, 63, 64, 67, 73, 74], 2 indicating PP (primiparous) [61, 76], and another 2 specifying MP (multiparous) [55, 74] as the predominant status. On the other hand, 23 articles reported gravida status, with a majority (12 out of 23) articles reporting most of their patients as PG (primigravida) [32, 33, 35–37, 39–41, 45, 47, 62, 65]. Moreover, the majority of the patients from 2 articles were G2 (gravida 2) [38, 85], and from 4 articles were MG (multigravida) [53, 54, 56, 71].

### Risk of bias of included studies

Upon quality assessment using the NHLBI tool for observational studies, 38 studies received a good rating [15, 31–39, 41, 42, 44–46, 48, 51, 53–55, 57–63, 65, 67–69, 71, 72, 74, 75, 77, 79, 80], 3 a fair rating [47, 50, 78] and 13 studies received a poor rating [40, 43, 49, 52, 56, 64, 66, 70, 73, 76, 81–83]. Quality assessment is depicted in Supplementary Table 3.

### Gestational hypertension

**Fig. 3:**
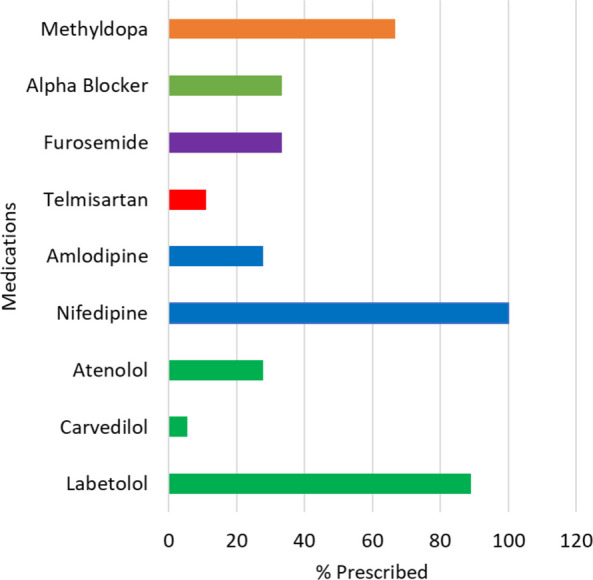
Articles reporting medications for gestational hypertension

Figure 3 shows the prescribing patterns for gestational hypertension (GH). In terms of medical interventions, for the management of GH, Calcium Channel Blockers (CCBs) were the most frequently prescribed classes of medication and were mentioned in all the 18 relevant studies [31–33, 35–37, 39, 42, 43, 45–47, 50, 53, 68, 71, 79, 86], with labetalol (88.89%) and nifedipine (88.89%) being the most common prescribed drugs. Moreover, 67% (12/18) studies reported the use of methylopa [32, 35–37, 39, 40, 42, 46, 50, 68, 71, 79]. β-blockers included atenolol used in 5 (27.78%) studies [35, 36, 47, 68, 71] and carvedilol used in 1 (5.56%) [31]. Among other CCBs, amlodipine was mentioned in 5 (27.78%) articles [31, 36, 40, 47, 50]. Additionally, a diuretic, furosemide was reported in 6 (75%) studies. Alpha blockers were reported by 6 articles (33.33%) [31, 35, 39, 40, 46, 68]. Telmisartan prescription was reported in 2 articles [36, 40].

### Pre-eclampsia

**Fig. 4:**
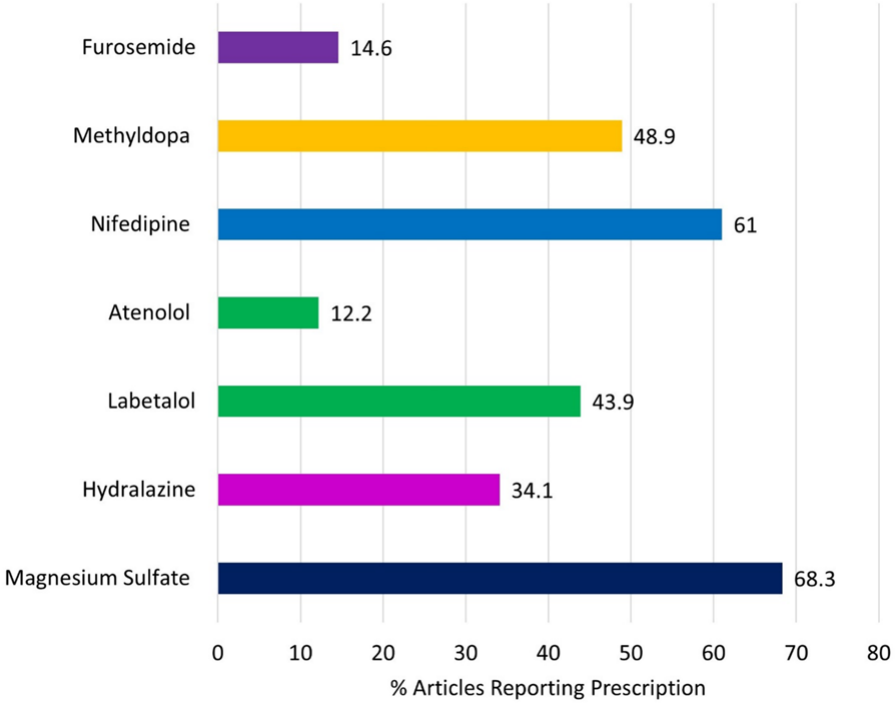
Articles reporting medications for pre-eclampsia

For pre-eclampsia as seen in Fig. 4**,** out of the 41 relevant articles, magnesium sulphate was reported in 28 (68.29%) articles [15, 34, 36, 38, 43, 46, 47, 51, 52, 57–59, 63–65, 67, 70–73, 75, 76, 78–83], nifedipine in 25 articles (60.98%) [15, 32, 34–43, 46, 47, 53, 54, 59, 68, 69, 71, 72, 75, 77, 79, 80], methyldopa in 20 articles (48.78%) [15, 32, 34–39, 42, 46, 54, 59, 67–69, 71, 77, 79, 80, 82], labetalol in 18 articles (43.90%) [32, 34, 35, 37–43, 47, 53, 69, 71, 76, 77, 79, 83], hydralazine in 14 articles (34.15%) [15, 54, 59, 60, 64, 67–69, 71–73, 77, 79, 83], furosemide in 6 articles (14.63%) [32, 34, 39, 43, 47, 69], and atenolol in 5 articles (12.20%) [32, 34, 36, 47, 68].

### Eclampsia

Out of 33 articles, 28 (84.85%) mentioned the use of MgSO_4_. Eight articles cited the utilization of MgSO_4_ in combination with other drugs, specifically Phenytoin in 2 articles [36, 68], Labetalol in 1 article [47], Diazepam in 2 articles [15, 68] and Nifedipine in 2 articles [47, 68]. Additionally, one article also discussed the inclusion of Benzodiazepine or Carbamazepine in combination with MgSO_4_ and Phenytoin [36]. Similarly, 3 more articles [47, 57, 81] recommended MgSO_4_ as the medication of choice for preventing seizures during eclampsia.

Only 3 articles mentioned the specific MgSO_4_ treatment regimen administered for Eclampsia. All three [45, 52, 83] mentioned the Pritchard regimen whereas one [52] mentioned the Zuspan regimen along with Pritchard being used as a treatment approach. 15 articles specified the mode of administration of MgSO_4,_ however, only 3 [45, 49, 68] suggested the preferred mode of administration as Intramuscular (IM). 7 articles [45, 49, 52, 64, 72, 79, 83] mentioned both Intramuscular and Intravenous (IV) as the mode of administration, while 4 articles [15, 69, 75, 76] only stated IV administration. Where both IM and IV were mentioned, one article clarified that IV administration required greater expertise [49].

Eight articles explored the reasons for under-utilization of MgSO_4_ for Eclampsia. Among the reasons mentioned, 5 articles [57, 59, 62, 70, 80] cited the unavailability of MgSO_4_ as a reason for not prescribing the medication. 2 [36, 62] emphasized inadequate seizure control and 1 [62] mentioned other contraindications but did not provide further clarification. Incorrect administration, lack of expertise, poor prescribing practice, and missed doses were identified by 5 articles [52, 57, 64, 66, 78] as reasons for low MgSO_4_ use.

Eight articles commented on the parameters used for therapeutic drug monitoring during the administration of MgSO_4_ [36, 64, 67, 72, 73, 75, 78, 83].

Table 3 describes the country-wise prescribing patterns for Phenytoin and Diazepam. Among the 33 articles mentioning eclampsia, phenytoin was prescribed in 5 (15.15%) [36, 52, 62, 68, 81]. Diazepam was prescribed in 11 articles, accounting for 33.33% of the articles [15, 52, 58–60, 62, 63, 65, 67, 68, 80].

**Table 3:** Prescribing pattern of Non-MgSO4 anti-convulsant for eclampsia

Author(s)	**Year of Publication**	**Country**	Diazepam	Phenytoin
1. Khedun et al. [68]	2000	South Africa	✓	✓
2. Ikechebulu et al. [60]	2002	Nnewi	✓	NR
3. Shaheen et al. [80]	2003	Pakistan	✓	NR
4. Lumbiganon et al. [81]	2007	Mexico and Thailand	NR	✓
5. Tukur et al. [58]	2009	Nigeria	✓	NR
6. Kidanto et al. [73]	2009	Tanzania	NR	NR
7. Abd El Aal et al. [75]	2011	Egypt	NR	NR
8. Sariem et al. [59]	2012	Nigeria	✓	NR
9. Kumar T et al. [36]	2013	India	NR	✓
10. Chaturvedi et al. [49]	2013	India	NR	NR
11. Kim et al. [15]	2013	Afghanistan	✓	NR
12. Ahadi et al. [67]	2015	Afghanistan	✓	NR
13. Oguntunde et al. [57]	2015	Nigeria	NR	NR
14. Devkota et al. [65]	2016	Nepal	NR	NR
15. Yousef et al. [78]	2017	Sudan	NR	NR
16. Ali et al. [83]	2018	Pakistan	NR	NR
17. Katageri et al. [52]	2018	India	✓	✓
18. Selvi et al. [47]	2019	India	NR	NR
19. Williams et al. [70]	2019	Bangladesh	NR	NR
20. Ansari et al. [66]	2019	Afghanistan	NR	NR
21. Maaløe1 et al. [72]	2019	Tanzania	NR	NR
22. Toure et al. [76]	2019	Senegal	NR	NR
23. Ngene et al. [69]	2020	South Africa	NR	NR
24. Tlaye et al. [65]	2021	Ethiopia	✓	NR
25. Getaneh et al. [62]	2021	Ethiopia	✓	✓
26. Billah et al. [71]	2021	Bangladesh	NR	NR
27. Malhamé et al. [79]	2021	Haiti	NR	NR
28. Godana et al. [63]	2021	Ethiopia	✓	NR
29. Pawar et al. [45]	2022	India	NR	NR
30. Blessy et al. [39]	2022	India	NR	NR
31. Meazaw et al. [64]	2022	Ethiopia	NR	NR

NR: Not Reported

✓: Prescribed

## Discussion

Our systematic review assesses the literature on prescribing patterns of medications for HDPs in LMICs. The first line medications according to most guidelines included labetalol and calcium channel blockers and were also the most frequently prescribed. MgSO_4_ was also reported in most studies for pre-eclampsia and eclampsia. These results illustrate the correct prescription of medication in most LMICs. However, some discrepancies were seen within studies from different countries and settings, likely due to the use of different guidelines or medication availabilities.

Most of our included studies were from Asia, particularly India. A possible reason for this could be due to more initiatives regarding pharmacovigilance in India [87]. Moreover, India and even other countries such as Nigeria and Ethiopia from where articles were retrieved have a history of WHO collaborations to facilitate hypertension control, the byproduct of which could be the increased number of publications [88].

The WHO has published guidelines on the management of non-severe hypertension (≥ 140/90 but < 160/110) during pregnancy [4, 9]. They emphasize the practicability of these guidelines in “all settings”, with an emphasis on standardizing care globally. The WHO recommends the use of either methyldopa or beta-blockers as first line agents in the management of non-severe hypertension, based on their safety profiles and availability in various countries [4, 9]. However, while labetalol was used extensively, our results indicate that methyldopa was less commonly used.

The NICE guidelines recommend labetalol and nifedipine to be used whereas SOMANZ supports nifedipine's role as a second-line medication [30, 89]. Whilst our findings show that nifedipine was used, as it was reported by all studies in LMICs.

The variations in prescribing patterns for gestational hypertension in LMICs could likely be due to the use of different guidelines, absence of institutional guidelines and lack of refresher training on optimal management. Although, international guidelines primarily from HICs such as NICE, SOMANZ, ISSHP, and ACOG are followed in LMICs [6, 30, 89], it is crucial to note that very few established institutional guidelines in LMICs, specify recommendations based on local population-based trials and available resources [90]. It should also be noted that out of all the guidelines, only ACOG, QLD, NICE, NVOG, SOGC and WHO discuss antihypertensive therapies [27], shedding light on the need for improved guideline reporting to allow low resource settings to keep pace with updated managements. Moreover, even with national guidelines in some countries like Afghanistan, inadequate training, and misperceptions regarding the importance of guided treatment play a role in the lack of adherence [15]. A study from Pakistan found similar reasons for subpar managementcontributing to reduced knowledge on management [91]. Therefore, with an absence of institutional guidelines and a handful of international guidelines to choose from, it generally depends on individual physicians to determine which guidelines they choose to follow [91].

Although, most of our included studies were from the tertiary care level, the few studies we did have from the primary care level found a lack of adherence to first-line treatment regimens [52, 70, 71]. Majority of pregnant women access primary health care services throughout their pregnancy, and it has been found that HDP cases in these settings are often detected late. Hence, future guidelines should not only be adapted by institutions in LMICs, but also be contextualized for multiple settings, especially primary care [11, 92].

For PE, our study found magnesium sulfate followed by nifedipine to be the most widely used drug. MgSO_4_ was the most used medication, however clinical practice guidelines have less certainty about recommending it for non-severe PE as seen in NVOG, ACOG and SOGC, albeit no guideline recommends against it [27]. In comparison, NICE guidelines have outlined IV hydralazine, labetalol and oral nifedipine as the first line for emergency treatment of preeclampsia [93].

Interestingly, only 34% of the included articles reported hydralazine use, suggesting an under usage of hydralazine for pre-eclampsia in LMICs. Hydralazine despite being frequently listed in national LMIC essential medicine lists (EMLs) according to a 2013 prevalence study [94], was not the most prescribed drug for preeclampsia. Further research needs to be conducted in LMICs to determine the extent of this disparity; however, possible reasons could be use of different guidelines, affordability, and accessibility for cardiovascular disease medications [95]. At the same time, randomized controlled trials which often form the evidence behind guidelines have had recent findings where intravenous hydralazine and oral nifedipine were found to be equally effective for acute hypertensive emergencies in pregnancy [96].

Other guidelines such as the ACOG recommend methyldopa and labetalol as first-line agents whilst beta-blockers and ACE inhibitors are not recommended [97]. Our study did find a prescribing pattern of atenolol, although minimal, in LMICs. Its use might be the result of controlling blood pressure outweighing the potential risks to the fetus. 

Most of the articles mentioned using magnesium sulfate (85%) for eclampsia. The consensus within the literature is that magnesium sulfate is no longer recommended as an antihypertensive, but instead considered the drug of choice for the prevention and treatment of eclampsia in all cases of severe preeclampsia [93, 98–103]. Very few articles highlighted the treatment regimens presenting further insight into the lack of detailed research within LMICs.

Amongst the articles discussing regimens, our result complied with previous literature which finds Prichard to be the more popular choice in resource-limited settings [104]. However, it is to be noted that the Pritchard regimen consisting of a high dose has shown multiple dose-related toxicities [105]. Thereby, low-dose regimens have been evaluated but despite signs of efficacy, simplicity and safety, lack of LMIC-specific studies and an absence of protocol standardization have hindered its incorporation into guidelines [106–109]. A recent study from 2023 further explored using a modified Zuspan regimen which could apply to developing countries facing cost and availability issues for procurement of magnesium sulfate and found no significant difference in seizure recurrence, thereby serving the possibility of using 12 h modification of the maintenance dose of Zuspan regimen for the management of severe pre-eclampsia and eclampsia. However, further studies considering the long-term effects of a reduced maintenance dose of MgSO_4_ on the neonates are required [110]. A study from Afghanistan found adequate supply in their facility but a lack of knowledge and clear service delivery guidelines prevented their use [15]. Therefore, medication delivery should be supplemented with refresher training to reinforce best practices.

Aside from magnesium sulfate, phenytoin (15%) and diazepam (33%) were also part of the prescription for eclampsia. Although magnesium sulfate is first line and recommended for eclampsia, other anticonvulsants are also used when magnesium sulfate is contraindicated or ineffective, or simply for the treatment of convulsions. The use of phenytoin in LMICs could likely be explained by its ability to reduce time to return to consciousness, which is particularly useful in hospitals with higher patient volumes [111]. In addition, the caesarean section rates have also been less with phenytoin compared to magnesium sulfate, requiring fewer skilled workers to manage the delivery, benefiting workforce-limited areas in LMICs [112]. Data from other LMIC studies have also shown a slight preference for diazepam in cases of eclampsia with two of the nine doctors mentioning regular use of diazepam [113]. However, spite ofbeing used as alternatives for eclampsia, magnesium sulfate has fared as a better preventer of seizure than all other alternatives [114, 115].

The unique challenges that healthcare providers experience in LMICs may contribute to differences in prescribing patterns for the management of hypertensive conditions during pregnancy. The way forward highlights the need for increased population-based studies within LMICs and their inclusion within international consensus guidelines. Moreover, institutions must practice pharmacovigilance and provide adequate refresher training to ensure proper adherence.

### Strengths and limitations

Our study is the first baseline assessment of prescribing patterns for HDPs in LMICs and the first of its kind to discuss the adherence of LMICs with management guidelines. This review serves as a snapshot of the medications utilized for the management of hypertensive disorders during pregnancy in low-middle income countries. It is essential to assess over time the evolution of these prescribing patterns to determine the effectiveness of interventions targeted at facilitating adherence to guidelines. Therefore, public health researchers may compare their results against our review in the future.

Our study, despite focusing on LMICs, many of which do not have English as a first language, only included English language articles, due to which local databases in local languages could have been excluded. In addition, despite some multicenter studies, the included articles are largely single-center based on small hospital-based studies leading to chances of sampling bias and limited generalizability. The prescriber of the medication was also not specified in all the studies and so future studies should include to better understand the healthcare setting. Other hypertensive disorders of pregnancy such as chronic hypertension or pre-pregnancy hypertension medication patterns were not recorded in our study due to a lack of standardized data pertaining to their management, and therefore should be evaluated in future studies. Lastly, due to the nature of our review, the holistic approach when a patient is prescribed medication that involves their past medical history and comorbid was not gauged in our study and the focus was purely on prescriptions. Detail in treatment regimens along with other indications is a gap in LMIC literature which if addressed, can lead to more detailed analytic studies in the future.

## Conclusions

Our review of literature shows that prescribers in LMICs are appropriately choosing the first line agents for HDPs. Slight differences between prescribing patterns in LMICs and international guidelines are seen, which could likely be attributed to a lack of resources, not being up to date with latest research and inadequate training. With the absence of institutional guidelines in many settings, it is evident that an intervention is required to re-evaluate guidelines appropriate to LMICs as well as a detailed investigation regarding patient outcome and medical services. Our review serves as a baseline assessment of current practices to enable targeted evidence-based policies.

## Data Availability

Most of the data presented is provided either in the form of tables or figures. Any remaining data is available from the corresponding author on reasonable request.

## Abbreviations

ACOG: American Congress of Obstetricians and Gynecologists
GH: Gestational Hypertension
HDP: Hypertensive Disorder of Pregnancy
HIC: High-Income Country
ISSHP: International Society for the Study of Hypertension in Pregnancy
LMIC: Low- and Middle-Income Country
MgSO_4_: Magnesium sulfate
NICE: National Institute for Health and Care Excellence
NVOG: Dutch Society of Obstetrics and Gynaecology
PE/E: Pre-eclampsia/eclampsia
QLD: Queensland Clinical Guideline
SOGC: Society of Obstetricians and Gynaecologists of Canada
SOMANZ: Society of Obstetric Medicine of Australia and New Zealand
WHO: World Health Organization
